# Enterosorbents in complex therapy of food allergies: a focus on digestive disorders and systemic toxicity in children

**DOI:** 10.3389/fimmu.2023.1210481

**Published:** 2023-10-13

**Authors:** Valentin P. Shichkin, Oleg V. Kurchenko, Elena N. Okhotnikova, Valentyna V. Chopyak, Domenico V. Delfino

**Affiliations:** ^1^ OmniFarma, LLC, Kyiv, Ukraine; ^2^ Department of Pediatrics, Children’s Infectious Diseases, Immunology and Allergology, Shupyk National Healthcare University of Ukraine, Kyiv, Ukraine; ^3^ Department of Clinical Immunology and Allergology, Danylo Halytsky Lviv National Medical University, Lviv, Ukraine; ^4^ Master in Musculoskeletal and Rheumatological Physiotherapy, Department of Medicine and Surgery, University of Perugia, Perugia, Italy

**Keywords:** food allergy, pseudoallergy, enterosorbents, detoxification, silicon dioxide, White Coal (Carbowhite), immunotoxicity

## Abstract

The review analyzes mechanisms and concomitant factors in developing IgE-associated allergic diseases provoked by food allergens and discusses clinical symptoms and current approaches for the treatment of food allergies. The expediency of using enterosorbents in complex therapy of food allergies and skin and respiratory manifestations associated with gastroenterological disorders is substantiated. The review summarizes the experience of using enterosorbents in post-Soviet countries to detoxify the human body. In this regard, special attention is paid to the enterosorbent White Coal (Carbowhite) based on silicon dioxide produced by the Ukrainian company OmniFarma.

## 1 Introduction

According to the World Health Organization (WHO), allergic diseases are the third most common after cardiovascular and oncological pathologies. Approximately 40% of the world population is allergic to drugs, household and industrial allergens, insect bites, food ingredients, etc. According to WHO forecasts, by the end of the 21st century, every second person will suffer from some form of allergy (1).

Allergic diseases are one of the most common somatic diseases of childhood. In recent years, the prevalence of allergic diseases in schoolchildren has increased, reaching 20% (2, 3). The formation of allergies in children is characterized by the stages of development of sensitization and the transformation of clinical manifestations, depending on the child’s age. In children with atopy, the allergy generally manifests in food allergies and atopic dermatitis already in early childhood. Later, 10-15% of children develop allergic rhinitis, and 40-43% develop bronchial asthma. The severe course of atopic dermatitis and atopic rhinitis is a risk factor for the subsequent development of bronchial asthma (2–5). Since this process is often triggered by early childhood food allergies, therapeutic and preventive measures aimed at timely detection and compensation of allergic manifestations in children caused by food components are relevant.

The increasing prevalence of allergic diseases among schoolchildren and the high prevalence of food allergies in early childhood are complex issues influenced by a combination of genetic, environmental, and lifestyle factors. While the exact causes are not fully understood, several potential contributing factors are discussed (2–5): 1) Allergies often have a genetic component. Children with a family history of allergies are at a higher risk of developing allergic diseases. 2) The hygiene hypothesis suggests that reduced exposure to infections and certain microbes in early childhood due to increased hygiene and reduced family size may lead to an improperly developed immune system resulting in allergies. 3) Changes in diet, such as increased consumption of processed foods and a decrease in the consumption of fresh fruits and vegetables, may influence the development of allergies. 4) Exposure to environmental factors like pollution and a lack of contact with animals and rural environments may impact immune system development and contribute to the rise in allergies. 5) Early life events, including the mode of birth (cesarean section vs. vaginal birth) and breastfeeding practices, can influence a child’s susceptibility to allergens. 6) The composition of the gut microbiota in early life can play a crucial role in immune system development. Changes in the gut microbiota due to factors like antibiotic use, diet, and hygiene practices may impact the risk of allergies. 7) Psychological stress and lifestyle factors, such as lack of physical activity or excessive screen time, may also influence immune system function and contribute to the development of allergies. 9) Vaccination practices and antibiotic use in early childhood may influence allergy development.

It’s important to note that these factors likely interact with each other, and the specific combination of influences can vary from one individual to another.

Food allergy is one of the types of allergic reactions that develops when eating certain foods that contain an allergen, which in turn causes an aggressive pathological response of the immune system, which manifests, in most cases in an immediate type I reaction, due to hypersynthesis and critical participation of IgE (4–6). Food allergy occupies a special place among allergic manifestations in terms of prevalence and peculiarities of its etiology, prevention, and treatment. It is one of the main reasons for developing allergic diseases in children (5, 7–10). Medical observations suggest that food allergy occurs in 6-8% of children under two years of age (60-94% of those cases occur in the first year of life), with a subsequent decrease in its prevalence to 2% among the adult population (4, 5, 10).

Food allergy symptoms are often presented through itching in the mouth and larynx, laryngeal edema, coughing for no reason, wheezing and shortness of breath, and pain during a conversation. Nausea, vomiting, and diarrhea may meanwhile occur in more acute cases. With food allergies, hives may appear due to a sharp influx of blood to the face. Urticaria, in turn, can provoke bronchial spasms, laryngeal edema, and arterial hypertension, leading to severe consequences, even death. Most patients have a combination of several symptom complexes (7–10). Prevalence of food allergies varies widely and ranges from 0.9 to 13% of all allergic reactions (8, 10).

One of the causes of allergic diseases is considered to be a sizable antigenic load on the body due to combined effects of natural and anthropogenic factors, in particular artificial pollutants, which also include bio-pollutants. In addition to exogenous factors, there are also endogenous factors, primarily burdened heredity. It has been established that 40% of people have a hereditary tendency to atopy and that this population is susceptible to adverse environmental conditions (1, 8, 10). Over time, these individuals develop immediate-type allergic hypersensitivity with elevated levels of IgE, i.e., atopic allergic diseases type I (1, 3, 11). Often there is a combined effect of several factors: an unfavorable environmental situation, occupational hazards, social conditions, etc., that primarily affect patients with a hereditary predisposition (1–3, 12).

Of the endogenous factors, concomitant diseases of liver, kidneys, alimentary canal, respiratory system, and skin also play an essential role in allergy formation. Analysis of physical status of allergic patients showed a high percentage (67.5%) of concomitant pathology, especially liver pathology (chronic hepatocholecystitis, cholelithiasis, biliary dyskinesia), the alimentary canal (intestinal dysbiosis, enterocolitis, helminthic invasion, etc.), kidneys, etc. (3–6, 10). These diseases play an essential role in pathogenesis of allergic disease relapses.

Impairment of barrier function of internal organs in chronic diseases facilitates entry of various exoallergens (drugs, dust, food components, etc.) and xenobiotics of industrial origin into the body and also deteriorates detoxification and elimination of these foreign substances from the body. As a result of significant antigenic stimulation of immunocompetent cells, hyperproduction of IgE occurs (primarily in individuals with hereditary atopy), increased synthesis of immune complexes with damage to the membrane of mast cells (blood basophils), and release of biologically active substances from them into bloodstream - histamine, serotonin, acetylcholine, etc. Accumulation of these substances in the body leads to development of endotoxicosis and aggravates the patient’s condition (3, 10–15).

Endotoxicosis is understood to be a multifactorial pathological process based on systemic tissue hypoxia with all its complex metabolic consequences. With endogenous intoxication, catabolism processes intensify, tissue alteration, liver and kidney failure, microcirculation disorders, and metabolic disorders occur. Exposure of cells to xenobiotics leads to changes in the properties of their membranes and disruption of intracellular homeostasis and metabolism. As a result of these pathological processes, toxins penetrate intercellular space and enter bloodstream. Regardless of how exoallergens enter the body, biologically active substances enter bloodstream and are distributed throughout organs and tissues affecting them either at the site of penetration or at the level of organs and systems. Toxic products of allergic reactions and various ecopathogens (in a native or transformed form) enter through liver, pancreas, and intestinal mucous secretions, into the digestive canal’s lumen, from where they can be reabsorbed into the blood. Having passed phases of biotransformation, xenobiotics as endogenous toxic substances are thus distributed between the blood, tissues, and enteric system (15–22).

Given the mechanisms of pathogenesis and toxic manifestations of allergic reactions, it is advisable to use detoxification methods in the complex treatment of patients with allergic diseases. From this point of view, enterosorbents, and in particular, enterosorbents based on silicon dioxide, which have been available in the Ukrainian and other post-Soviet markets for over 30 years, have proven themselves as safe and effective. These are affordable nutritional supplements and medicines that have universal sorption and detoxifying properties to address a variety of pathogenic intestinal microorganisms, food, helminthic, household and industrial toxins and allergens, circulating immune complexes, and inflammatory mediators (23–35) – all factors that play a critical role in sensitization and development of allergic reactions of various origins. These nonspecific properties of enterosorbents make it possible to be considered reasonable components in complex therapy of allergic diseases, which can significantly alleviate the course of these diseases and improve the life quality of sensitized patients.

## 2 Immunological mechanisms of food allergy

### 2.1 Role of gut microbiota

Dgestion and absorption of food products depend on the state of the neuroendocrine system, structure and function of the digestive and hepatobiliary systems, composition and volume of digestive juices, composition of the intestinal microflora, state of local immunity of the intestinal mucosa (lymphoid tissue, secretory immunoglobulins, etc.) and other factors. Usually, food products break down into compounds that do not have sensitizing properties (amino acids and other non-antigenic structures), and the intestinal wall is impermeable to non-digested products that have or may have, under certain conditions, sensitizing activity or able to cause pseudoallergic reactions (6, 8–10). Food allergens are digested and absorbed mainly in the small intestine, which is enriched by symbiotic microbiota. Thus, the intestinal cavity presents a homeostatic environment in which immune cells respond favorably to food allergens. However, the type and number of symbiotic microbiota in the gut may change and be affected by many external factors particularly, changes in dietary patterns, antibiotics use, breastfeeding, vaccines, pathogen exposure, etc. Experimental and epidemiological studies suggest that the gut microbiota composition is related to the clinical manifestation of food allergies and that early life (0–6 months) is a critical period for gut microbial colonization. Gut microbiota has been shown to affect the growth of immune tolerance to food antigens by modifying regulatory T cell (Tregs) differentiation, regulating basophil populations, and enhancing intestinal barrier function. Several gut microbial metabolites, such as short-chain fatty acids, secondary bile acids, and amphoteric polysaccharide A may directly or indirectly regulate Tregs differentiation, most of which express the retinoic acid receptor (RAR)-associated orphan receptor γt (RORγt) (36).

RORγt is a master regulator of interleukin (IL)-17-producing intestinal CD4+ T helper (Th17) cells (37, 38). This transcriptional factor is highly expressed also by IL-22-producing CD4+ Th cells and IL-17/IL-22-producing innate lymphoid cells (ILCs), in particular, subset group 3 (ILCs3) located in the intestine (39–43). Aryl Hydrocarbon Receptor (AhR) is another transcription regulator acting synergistically with RORγt (44, 45). The AhR can either directly regulate IL-22 gene expression and cytokine production or regulate the production and development of ILC3 and Th17 cells (42, 44–46). AhR ligands derived from gut microbiota are an essential component in initiating the transcription of IL-22 (46–48). Through IL-17- and IL-22-mediated signaling, RORγt and AhR can enhance the intestinal epithelial barrier, defend against pathogenic microflora, and regulate the balance of symbiotic gut microbiota which play a crucial role in the formation of intestinal immunity (38, 42–45).

Intestinal immune cells are mostly reserved in the gut-associated lymphoid tissues. However, a diverse array of immune cells (both, the innate and the adaptive immune system) are also present in the lamina propria and the layer of epithelial cells forming mucosa which is colonized by symbiotic gut microbiota (49–51). This provides easy communication signaling between gut microbiota and immune cells supporting the healthy microbiota balance and correct immunoreactivity and preventing dysbiosis and allergic reactions to food components which may damage the gut epithelial barrier and promote the expansion of pathogenic microflora. These highlights support a strong relationship between gut microbiota quality and food allergies or tolerance development, but the exact regulatory mechanism remains unclear.

Various factors, including dietary patterns, antibiotic usage, environmental factors, and early-life factors like breastfeeding, can impact the gut microbiota composition and development of food allergies. Antibiotic use in infancy can selectively deplete specific microbial species, potentially favoring the overgrowth of undesirable bacteria, affecting immune system maturation, and increasing the risk of allergies. A diverse microbiota is generally associated with a healthier immune system and reduced allergy risk. There are several therapeutic strategies proposed to prevent dysbiosis and gut microbiota restoring. Diets rich in a variety of fibers and nutrients can promote a diverse gut microbiota. Prebiotics (nondigestible fibers that feed beneficial gut bacteria) and probiotics (live beneficial bacteria) can help maintain a balanced gut microbiota. These supplements have been explored as potential interventions to reduce the risk of food allergies, although more research is needed to determine their effectiveness (49, 50). Breast milk contains beneficial microbes and prebiotic compounds that support the growth of beneficial gut bacteria in infants. Breastfeeding is associated with a reduced risk of food allergies, likely due to its positive impact on the infant’s gut microbiota (49). Certain microbial metabolites, such as short-chain fatty acids, have immunomodulatory effects. Bacterial metabolites can promote the development of Tregs, which help maintain immune tolerance. Therapies aimed at boosting the production of these metabolites, such as dietary interventions or microbial supplementation, are being explored (51). As each individual’s gut microbiota composition is unique, personalized approaches to food allergy prevention and treatment may become more common. These approaches might involve gut microbiota profiling and tailoring interventions based on an individual’s specific microbial makeup. Strategies to reduce environmental factors that disrupt the gut microbiota, such as reducing unnecessary antibiotic use and promoting a cleaner but microbiota-friendly living environment, may be explored. Finally, we consider enterosorbent use as a beneficial approach to support the normalization of symbiotic gut microbiota and prevent the growth of pathogenic and opportunistic microflora and thus reduce risks of food allergy development. While specific mechanisms through which enterosorbents improve the balance of the gut microbiota are absent, the symbiotic microflora colonizing the mucosa of the intestine is less accessible for nonspecific adhesion in contrast to pathogenic transitory bacteria entering the intestine from the external environment and directly contacting with an enterosorbent. As a result, pathogenic bacteria are fixed by enterosorbents and naturally excreted from the intestine. A similar mechanism apparently also operates in the endogenous opportunistic microflora, if it persists in the intestine and has not yet colonized unspecific places for it.

It’s important to emphasize that while these therapeutic avenues hold promise, they are still areas of active research, and more clinical trials and studies are needed to establish their safety and effectiveness fully. More details about the correlation between the gut microbiota and immunological mechanisms of food allergies and tolerance and gut microbiota normalizing strategies are provided in recent reviews (49–51).

### 2.2 Role of IgE

Food allergies most often develop according to general mechanisms of IgE-mediated type I hypersensitivity, pathogenesis of which is associated with hereditary hyperproduction of IgE or due to allergic sensitization to various allergens, including some food components (1, 6). In this case, a patient synthesizes IgE antibodies to specific epitopes of food allergens (4). In genetically predisposed individuals, exposure to allergens leads to an increase in specific IgE that can bind to effector cells through a high-affinity receptor known as FcεRI, expressed by blood basophils and mast cells in various organs, including skin, digestive and respiratory tracts. As a result of complex formation and receptor activation, basophils and mast cells secrete proinflammatory mediators and cytokines, which develop inflammation and allergy symptoms (4, 6, 11, 12, 19). IgE is very short-lived (about one day) when free in plasma, but IgE bound to the FcεRI receptor forms pathogenic immune complexes and can remain attached to tissue cells for weeks or even months. Moreover, binding to the FcεRI receptor, IgE increases cell survival and receptor activation. Contact with a specific allergen causes the release of pharmacologically active mediators that are stored in mast cell granules and blood basophils, which leads to clinical manifestations of type I hypersensitivity (6, 13, 14).

### 2.3 Role of T helper type 2 cells

**Figure 1:**
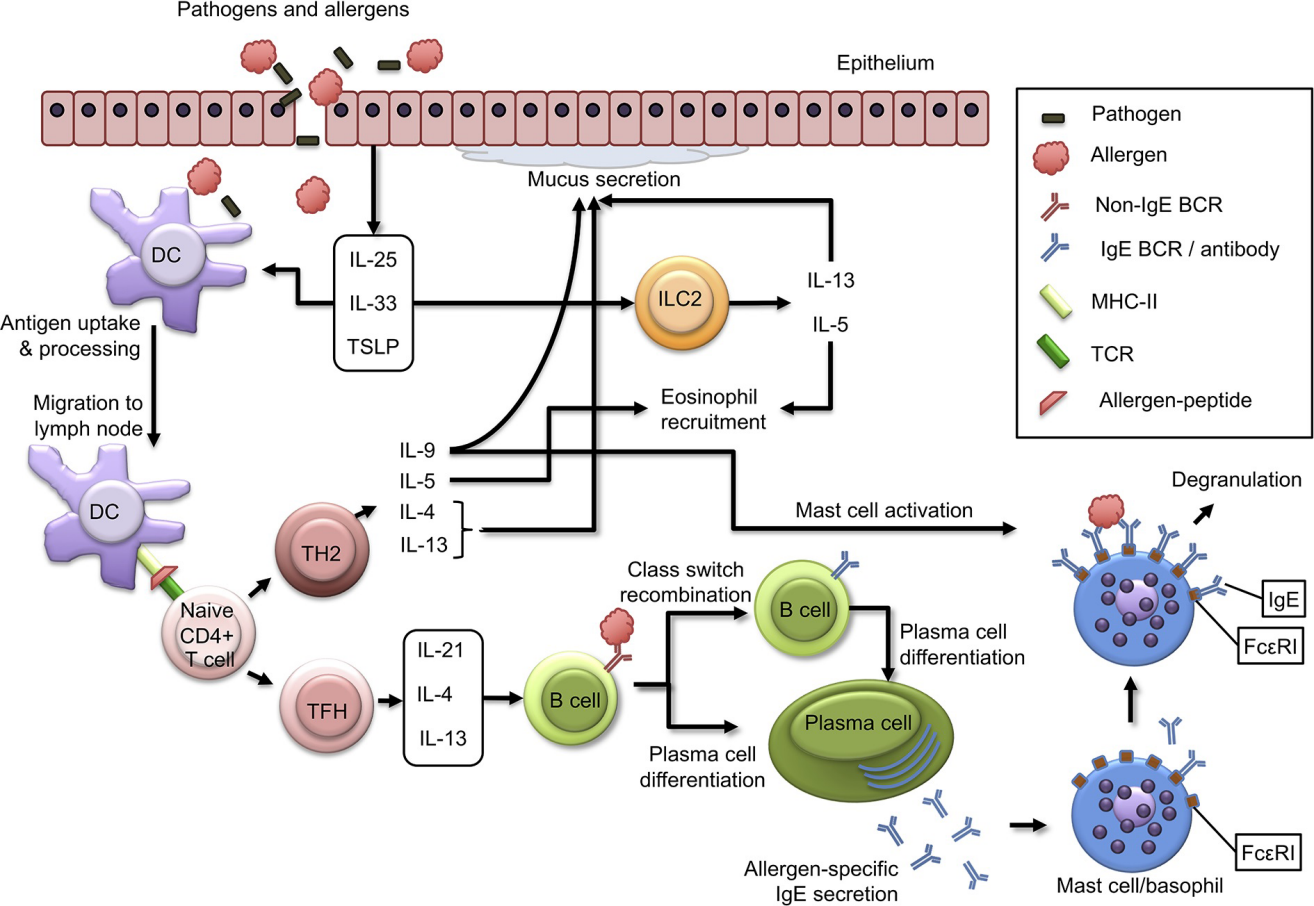
Immunological mechanisms of allergic sensitization. Description in the text. BCR, B Cell Receptor; DC, Dendritic Cell; IL, Interleukin; ILC2, Innate Lymphoid Cell Type 2; MHC-II, Major Histocompatibility Complex Type II; TCR, T Cell Receptor; TFH, T Follicular Helper Cell; TH2, T Helper Cell Type 2. Adopted from Schoos et al., 2020 (6); this is an open-access article distributed under the terms of the Creative Commons Attribution License (CC BY).

Allergic diseases result from a distorted immune response of Th2 cells to environmental antigens or allergens. In the case of food allergy, these allergens are usually food proteins causing predominantly IgE-mediated allergy (4–6, 8–10). The first step in allergy development is allergic sensitization, during which allergen-specific T and B cells activate, form clones, and differentiate (52). Allergic sensitization can occur through various routes of exposure to allergens. In type I hypersensitivity, allergens are initially presented to antigen-specific CD4+Th2 cells (53), which stimulate B cells to produce IgE antibodies, which are also antigen-specific (6). During IgE sensitization, antibodies bind to FcεRI on the surface of mast cells localized in tissues and blood basophils (12–14). Repeated exposure to the same allergen leads to binding of IgE on sensitized cells, resulting in degranulation and secretion of preformed pharmacologically active mediators, first of all, histamine (6, 14). All this happens as an immediate reaction that develops within a few seconds. The late response caused by induced synthesis and release of leukotrienes, chemokines, and cytokines by activated mast cells allows the recruitment of other leukocytes, eosinophils, basophils, and Th2-type lymphocytes to the site of allergic inflammation (**Figure 1**).

### 2.4 Role of dendritic cells

Most of the knowledge about mechanisms of food allergy and tolerance to food allergens comes from mouse models. Oral exposure usually induces tolerance but may also lead to sensitization, especially when adjuvants are present that disrupt the natural barriers of the alimentary canal, such as staphylococcal enterotoxin B (54). However, exposure to food allergens through the skin and respiratory tract can also lead to body sensitization (20–22). The association between food allergy and skin barrier impairment is also illustrated by high prevalence of food allergies among patients with atopic dermatitis, especially in its severe forms (20, 22).

Dietary proteins can pass through the intestinal epithelial barrier via transcytosis, paracellular diffusion, or endocytosis with help of microfold (M) cells (55, 56). In addition, intestinal epithelial cells can express MHC-II molecules and thus can directly present allergenic peptides to CD4+ T cells in the intestine (57, 58). Dietary proteins can also be taken up via transluminal processes by CX3CR1+ antigen-presenting DCs, which can select intestinal antigens by spreading transepithelial dendrites into the intestinal lumen. It is hypothesized that these CX3CR1+ DCs do not appear to migrate and fail to activate naive T cells, remaining in the intestinal epithelium. However, these cells can carry antigens to migrating CD103+ DCs in the intestinal mucosa (59, 60). A healthy response to food antigens is characterized by immune tolerance, mediated by antigen presentation in the gut by DCs. Peyer’s patches of the intestine are enriched in CD11c+ CD103+ DCs. After antigen uptake, these DCs can migrate to local lymph nodes, where they perform classical functions of DCs and control adaptive immune responses to food antigens (57–60).

Skin CD11b+ DCs and Langerhans cells are central players in induction of tolerance to allergens in the skin (61–63). Because of tissue damage and inflammation, epithelial cells produce Th2-inducing cytokines, IL-25, IL-33, and thymic stromal lymphopoietin (TSLP). These cytokines are primary regulators of type 2 immunity as they act on DCs to induce a Th2 cell response and activate innate lymphoid cells type 2 (ILC2), producing IL-13 and IL-5 (64). IL-33 is critical for activating the OX40 ligand (OX40L) on DCs in a mouse model of oral sensitization using cholera toxin, resulting in differentiation of naive Th2 cells (65). Allergens and pathogens that pass through cutaneous or epithelial mucosal barriers are captured and processed by DCs. When DCs exposed to epithelial-derived cytokines, they take up the allergen and migrate to the draining lymph nodes, where they present allergen peptides on MHC class II molecules to naive T cells leading to their clonal expansion, which, in turn (depending on costimulatory and cytokine signals) can differentiate into Th2 or TFH (T follicular helper) cells. Th2 cells become polarized towards producing type 2 cytokines, IL-4, IL-13, IL-5, and IL-9, and act as effector cells that control many aspects of allergic inflammatory responses.

### 2.5 Role of THF cells

While Th2 cells have long been considered critical for induction of IgE production by B cells, it has recently become apparent that IgE production requires interaction of B cells with TFH cells rather than with Th2 (66, 67). A recent study indicated that the production of high-affinity anaphylactogenic IgE antibodies depends on a subpopulation of TFH cells called TFH13 cells that secrete IL-13 in addition to IL-4 and IL-21 (66). Through the secretion of these mediators, TFH cells stimulate B cells to differentiate into allergen-specific IgE-producing plasma cells that can bind to surface FcεRIs receptors on basophils in blood and mast cells localized in tissues. Upon repeated exposure to the allergen, cross-linking of these FcεRIs receptors with allergen-specific IgE leads to degranulation of mast cells and basophils involved in Th2 response and a type I hypersensitivity reaction, culminating in clinical phase of the manifestation of food allergy (5, 6, 15, 17, 36, 52, 68, 69).

### 2.6 Role of cytokines

IL-4 and IL-13 are structurally and functionally similar cytokines playing a central role in allergic inflammation - they induce a switch in synthesis of immunoglobulin classes to IgE recombination, smooth muscle cell contraction, goblet cell hyperplasia, and mucus production (15). IL-5 also significantly contributes to allergic inflammation by recruiting eosinophils (68, 69). While IL-5-mediated eosinophilic inflammation has been convincingly demonstrated in some forms of asthma, it appears to be much less so in IgE-mediated food allergy (70). Interestingly, allergen-specific IL-5+ Th2 cells were found only in allergic patients with eosinophilic gastroenteritis. At the same time, peanut allergy is associated with a predominant IL-5+ Th2 cell response, indicating that heterogeneity in Th2 responses may contribute to IgE-mediated inflammation of the digestive system with predominance of eosinophilia (68). IL-9 contributes to occurrence of allergic diseases through stimulation of mucus secretion and release of chemokines by epithelial cells, as well as activation of mast cell proliferation (17, 71) (**Figure 1**). Food allergy can sometimes occur through a cell-mediated mechanism, leading to delayed symptoms or a chronic process. An example of such allergy is food-induced enterocolitis syndrome, which develops as a result of production of cytokines by T cells (68, 70, 72). Sometimes food allergies can develop in response to some food additives, especially azo dyes, in which case the latter act as haptens and, forming complexes with a protein, such as serum albumin, become fully-fledged allergens (6, 8, 10, 16). Mechanisms of formation of true food allergies have however yet to be thoroughly studied.

## 3 Associated factors of food allergy

Many external agents, including allergens, enter the digestive system. Due to unique properties of the mucosa of the digestive canal, a barrier exists in the intestine that serves to prevent penetration of allergens into the blood, including dangerous microorganisms, viruses, and toxins. Low acidity (pH) of gastric juices and presence of proteolytic enzymes both contribute to destruction of protein allergens, which in turn leads to loss of their allergenic properties (7–9, 19, 50, 73). Immune cells meanwhile protect the body from foreign agents and increase barrier properties of the intestine. Failure of the immunological defense program in the intestine’s immune system leads to increased permeability of the digestive canal mucosa for allergens and food allergies. This failure often manifests itself in overproduction of IgE antibodies and inability of the body to defend against invasion of antigens (4, 8, 10, 74). The antigen thus causes an alternating sequence of reactions: burning in the mouth, vomiting, abdominal pain, diarrhea, etc. When it enters the bloodstream, the allergen can cause drop in pressure, rash or eczema on skin, and bronchospasm in lungs (7–10). Almost any food product can become an allergen and cause development of food allergies. Protein products containing animal and vegetable proteins have more pronounced sensitizing properties, although there is no direct relationship between protein content and allergenicity of products (10, 75).

Of the endogenous factors, a significant role in formation of allergies is concomitant diseases of the liver (chronic hepatocholecystitis, cholelithiasis, biliary dyskinesia), the digestive canal (intestinal dysbiosis, enterocolitis, helminthic infestations, etc.), kidneys, respiratory system and skin. These diseases play an essential role in pathogenesis of relapses of hypersensitivity, both with and without an immunological mechanism of development (9, 16, 51). Presence of intestinal dysbiosis, even in subcompensated forms, often results in presence of products of incomplete digestion in the intestine. These, in turn, support clinical manifestations of food allergies since they can be allergens and, at the same time, enhance inflammatory changes in the digestive canal mucosa due to direct irritating effects (9, 54, 58, 72, 74).

Functional immaturity of the immune and digestive systems, insufficient production of digestive enzymes, deficiency of beneficial microflora, and intestinal infections, all play an essential role in formation of food allergies in children. The risk of a food allergy developing in a child may increase in presence of toxicosis, allergy, infection (helminthic, bacterial, fungal), and associated diseases, as well as being the result of an unbalanced diet during the mother’s pregnancy and during breastfeeding. Allergic sensitization of a child may be influenced by the nature of feeding (natural or artificial), types and timing of introduction of complementary foods, drug therapy, and allergens that enter the body from the environment. In addition, genetic predisposition to food allergies is of great importance. If neither parent has an allergy, then allergies are likely to occur in 4-10% of children; if one of the parents has allergies, then this probability increases to 25-50%; if both parents have allergies, then the probability of the child having an allergy increases to 40-80% (6, 8–10). A special low-allergenic diet currently is however not recommended for healthy people and pregnant and lactating women with a hereditary predisposition to atopy. On the contrary, it is believed that presence of allergens in the blood and milk of the mother contributes to development of tolerance to these food allergens in the child (76–78). However, exposure to epigenetic factors during fetal development and the first thousand days of life after birth remains a high-risk factor for child sensitization (79).

## 4 Non-allergic hypersensitivity (pseudoallergy)

With regard to allergic reactions to food components, it is important to distinguish between two main types of food allergy: true food allergy and pseudoallergy (non-allergic hypersensitivity). Non-allergic food hypersensitivity is clinically similar to an immediate type allergic reaction but it develops without participation of basic immunological mechanisms, such as IgE sensitization and Th2 response. Pseudoallergy differs from other reactions associated with food intolerance (defects or absence of digestive enzymes, psychoemotional intolerance) in that the same mediators are involved in its manifestation as in true food allergies (histamine, leukotrienes, prostaglandins, cytokines) albeit they are released from allergy target cells in a nonspecific way, that is, without the participation of IgE or other allergic antibodies. In this way, pseudoallergy is a result of the direct impact of food substrate antigens on target cells, in particular, mast cells, and, indirectly, with the activation of several biological systems by the antigen, such as the kinin system, the complement system, etc. in the absence of the immunological link of allergic sensitization (4, 8–10, 70).

Among the mediators of pseudoallergy, histamine plays a unique role. Many factors provoke development of food pseudoallergy: excessive intake of histamine into the body with foods rich in histamine, tyramine, and histamine liberators; excessive formation of histamine from food substrates; increased absorption of histamine with functional insufficiency of the digestive system mucosa; increased release of histamine from target cells; violation of the synthesis of prostaglandins, and leukotrienes (8, 9, 80).

Diagnosis of non-allergic hypersensitivity is based on the clinical pattern, dynamics of the course, and response to the elimination of pseudoallergy causes (80–83). True allergic reactions to food allergens are detected in approximately 35% of cases and pseudoallergic in 65% (9, 10, 12, 70).

Development of food allergies or pseudoallergies provokes disorders common to adults and children. It results in an increase in the permeability of the intestinal mucosa, pancreatic insufficiency, enzymopathy, inflammation of the biliary tract, intestinal dyskinesia, etc. Disorderly eating and rare or frequent meals meanwhile lead to impaired stomach secretion, gastritis, mucus hypersecretion, and other disorders contributing to development of food allergies or pseudoallergies (9, 10, 81, 84, 85). When digestive and hepatobiliary systems function correctly, food products supplied through the enteral route do not result in sensitization. Often the reason for pseudoallergies in food is not the foods themselves but various chemical additives introduced to improve taste, smell, and color and ensure shelf life (83).

Polymorphism of pseudoallergic skin rashes to food varies from urticaria (10–20% of the examined persons), papular eruption (20–30%), erythematous, and macular (15–30%) to hemorrhagic and bullous rashes (18, 19, 80, 82, 83). However, dividing food allergies into true and pseudoallergy is somewhat arbitrary. The same patient may develop hypersensitivity reactions to foods due to involvement of specific immune mechanisms and non-immune reactions. In addition, with a true food allergy, the patient may develop pseudo-allergic reactions to food over time, which aggravates the severity of the disease, making diagnosis difficult, and reducing the effectiveness of treatment.

## 5 Clinical manifestations of food allergy

### 5.1 Diversity of manifestations

**Figure 2:**
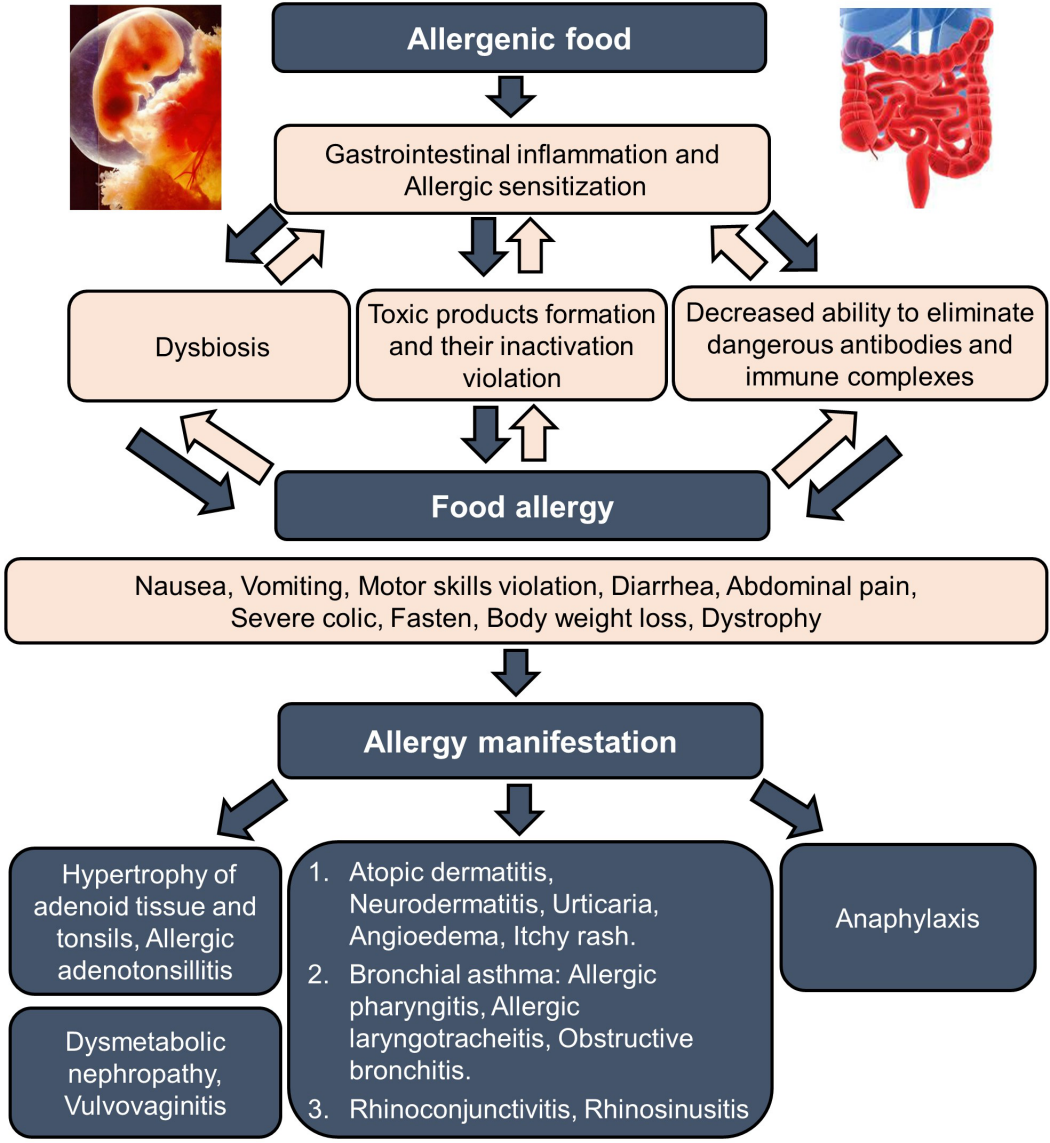
Food allergy formation and manifestation. The formation of food allergy in children to food components begins with a hereditary predisposition of parents to allergic diseases and intrauterine sensitization of the fetus (stage 1). These prerequisites are manifested in the first six months after birth as a food allergy with primary gastrointestinal and skin manifestations (stage 2). Damage to the alimentary canal provokes further sensitization to other allergens, including food. As a result, the food allergy progresses further, encouraging the development of atopic dermatitis and neurodermatitis, the maximal manifestations observed in the child age before 1-year-old (stage 3). After that, a progressing decrease in the allergic reaction to food allergens can be observed; however, the phenomena of bronchial obstruction (beginning of bronchial asthma) and allergic rhinitis may increase, which reach their pics to the ages 7-8 and 10-12 years, respectively (stage 4).

Clinical manifestations of food allergies are varied from gastrointestinal disorders to respiratory and skin manifestations, such as urticaria, angioedema (Quincke’s edema), and dermatitis. There are systemic and local allergic reactions that occur after exposure to food allergens, and their severity ranges from mild to extremely severe, such as in anaphylactic shock. A variety of conditions associated with food allergies have been described in children, among which the most common are atopic dermatitis, neurodermatitis, urticaria, angioedema, itchy morbilliform rashes, as well as lesions of the digestive system, which are manifested by nausea, vomiting, dysmotility, diarrhea, and abdominal pain, severe colic, constipation, weight loss, dystrophy. Less common are bronchial obstruction, laryngeal edema, rhinoconjunctivitis, and vulvovaginitis. Most patients have a combination of several symptom complexes (4, 5, 7–10, 19–22). **Figure 2** presents the main stages of food allergies development and its manifestations.

The earliest and most typical manifestation of a true food allergy is the development of oral allergy syndrome. After ingesting a particular food allergen, these patients develop swelling and itching in the lips, tongue, pharynx, and hard or soft palate (72). This syndrome is caused by cross-reactivity between certain types of food and pollen allergens. For example, a patient allergic to tree pollen may develop oral syndrome after eating apples, fresh carrots, peaches, cherries, and hazelnuts (9, 86, 87).

The most severe manifestation of food allergies is anaphylactic shock. Anaphylactic shock in food allergies differs in the speed of its development (from a few seconds to 4 hours), and the severity of the course impacting the likelihood of mortality, which ranges from 20 to 70%. Among the symptoms observed in anaphylactic shock, skin manifestations (84%), cardiovascular (72%) and respiratory symptoms (68%), are notably present. Anaphylaxis can also develop in the absence of skin manifestations. Respiratory and cardiovascular symptoms are however potentially life-threatening. Respiratory symptoms are more common in children, and cardiovascular symptoms are dominant among adults. Digestive symptoms such as nausea and vomiting may also occur. Biphasic anaphylactic reactions can occur in 20% of all cases. They usually appear 4 to 12 hours after the first symptoms and may be more severe. Delayed or insufficient administration of adrenaline (epinephrine) or erroneous administration of glucocorticosteroids may increase the risk of biphasic anaphylactic reactions (4, 8, 9, 88, 89).

Unlike true food allergies manifested by anaphylaxis, pseudoallergies can manifest as non-immunological anaphylaxis, known as anaphylactoid shock. Food-induced non-immunological anaphylaxis may resemble anaphylactic shock in clinical symptoms but differs in that it may lack polysyndromicity and a favorable prognosis. In non-immunological anaphylaxis, symptoms are observed mainly from one of the body systems, for example, drop in blood pressure or loss of consciousness. Still, all other parameters (skin, mucous membranes, breathing, etc.) are unchanged. The prognosis for non-immunological anaphylaxis is therefore favorable. With the timely appointment of adequate symptomatic therapy, the clinical response to treatment is observed quickly, normally in first hours of treatment (8–10, 70).

### 5.2 Gastrointestinal manifestations

Clinical manifestations of IgE-mediated diseases of the digestive system usually occur in combination with skin lesions and manifest in a variety of symptoms (vomiting, nausea, pain, diarrhea). In most children in first years of life, atopic dermatitis is a consequence of food allergies. 89-94% of children with atopic dermatitis caused by food allergens are diagnosed with the gastrointestinal allergy. In these children, especially in the first year of life, gastrointestinal allergies have the character of allergic enteropathy or allergic colitis. It most often has such manifestations as abdominal pain (colic), flatulence, regurgitation, and vomiting. Repeated exposure to food allergens leads to chronic inflammation, itching, and scratching, followed by clinically significant skin lesions (4, 5, 9, 10, 84). Any part of the digestive canal can be involved in the pathological process - from the oral cavity to the rectum. The most common gastrointestinal clinical manifestations of food allergies include vomiting, colic, anorexia, constipation, diarrhea, eosinophilic esophagitis, and allergic enterocolitis (9, 72, 74, 84, 90). Clinical manifestations are diverse and often nonspecific- infantile intestinal colic, regurgitation, vomiting, gastroesophageal reflux, eosinophilic esophagitis, gastritis, gastroenteritis, and proctitis. Chronic constipation resistant to conventional therapy, as well as malabsorption syndrome or protein-losing enteropathy, may also occur. Vomiting can occur from a few minutes to 4–6 hours after food ingestion and is often persistent. Vomiting is mainly associated with the spastic reaction of the pylorus when a food allergen enters the stomach. Eosinophilic esophagitis during endoscopy is detected in 15% of all patients who complained of dysphagia (10, 90). In 14–75% of cases, eosinophilic esophagitis is accompanied by other manifestations of allergy (bronchial asthma, atopic rhinitis, atopic dermatitis), and some patients have a history of IgE-mediated food anaphylaxis (8–10, 68, 69, 90). A combined lesion of several parts of the digestive system is possible (7–10, 18, 19, 72, 74, 75, 81, 84, 85). Symptoms of damage to the digestive system in patients with atopic dermatitis and allergic rhinitis may also indicate the role of food allergies in the etiopathogenesis of these diseases.

Allergic colicky abdominal pain can occur immediately after a meal or several hours later and is caused by smooth muscle spasms of the intestine associated with specific (true allergy) or nonspecific (pseudoallergy) liberation of allergy mediators. Abdominal pains are usually intense, sometimes displaying themselves as “acute abdomen,” accompanied by decreased appetite, mucus in the stool, and other dyspeptic disorders. Stool disorders, such as constipation and diarrhea, are also common manifestations of food allergies. Frequent, loose stools after ingesting a causally significant food allergen are among the most common clinical symptoms of food allergy in adults and children, especially in milk allergy (7, 9, 19, 72, 84, 85).

Allergic enterocolitis with food allergies is characterized by severe pain in the abdomen, flatulence, and loose stools with the discharge of vitreous mucus, which contains many eosinophils. Patients with allergic enterocolitis complain of extreme weakness, loss of appetite, headache, and dizziness (9, 12, 13, 73). Histological examination of patients with allergic enterocolitis reveals hemorrhagic changes, pronounced tissue eosinophilia, local edema, and mucus hypersecretion (7, 9, 68, 72, 81, 84, 85).

With food allergies, the intestinal microflora changes qualitatively and quantitatively due to the existing allergic inflammation and disturbances in the processes of digestion and absorption - frequency of dysbiotic disorders in children with food allergies reaching 94% (18, 19, 81, 84). Dysbiotic states create the conditions for implementing the virulent action of opportunistic pathogens. Putrefactive or fermentative flora and fungi, mainly of the genus Candida, develop abundantly, and microorganisms not typical for normal microflora are present (58, 84). Inflammation in various parts of the digestive system leads to impaired enzymatic activity, parietal and membrane absorption, and changes in microbiota. These changes in turn lead to increased formation of toxic products and their impaired inactivation, including a decrease in the ability to eliminate antibodies, and antigen-antibody complexes, which worsen the condition of patients with allergies and stimulate transition of the disease to more severe forms (4, 5, 8–10, 84, 88, 89).

### 5.3 Skin manifestations

Skin manifestations are the most common symptoms of food allergies in adults and children. In children under one year old, one of the first signs of food allergies can be persistent diaper rash, the appearance of perianal dermatitis, and perianal itching that occurs after feeding. Localization of skin changes in food allergies is different, but more often, they appear on the face and around the mouth, and can spread across the entire skin’s surface. At the onset of the disease, there is a clear connection between skin exacerbations and intake of a causally significant food allergen. Over time, allergic changes in the skin become persistent and constantly relapse, which makes it difficult to determine etiological factors. For true food allergies, most typical skin manifestations are urticaria, angioedema, and atopic dermatitis (5, 9, 10, 20, 21, 53, 91).

### 5.4 Respiratory manifestations

Allergic rhinitis in food allergies is characterized by presence of profuse mucus-watery discharge from nose, sometimes nasal congestion, and difficulty in nasal breathing. Rhinoscopy reveals swelling of the nasal concha mucosa, which has a pale cyanotic color. Often, along with rhinorrhea or swelling of the mucosa, patients sneeze and itch skin around or inside nose. The most frequent cause of allergic rhinitis in patients with food allergies is fish and fish products, crabs, milk, eggs, honey, etc. (4, 5, 9, 10).

### 5.5 Bronchial asthma

According to most researchers, the role of food allergens in developing bronchial asthma is small. Clinical manifestations of food allergies in the form of asthma attacks are observed in approximately 3% of cases (8–10, 19, 22, 92).

## 6 Treatment of food allergy

Main principles of treating food allergies are an integrated approach with phased-in therapy aimed at elimination of allergy symptoms and prevention of exacerbations. To reach optimal results, the treatment should include drug therapy, elimination therapy, environmental control, allergen-specific immunotherapy (3, 6, 8–10, 93–95), and nonspecific approaches, such as enterosorption (24, 27, 32, 33). The most promising treatment method being studied is allergen-specific immunotherapy (AIT), which primarily includes oral immunotherapy (OIT), sublingual immunotherapy (SLIT), and epicutaneous immunotherapy (EPIT) approaches (96–103). Additionally, there are currently actively studied adjunct and alternative treatment methods, such as use of IgE inhibitors, probiotics, and early introduction of allergenic foods in infancy (93–95, 97, 104–106). However, typical treatments still include food allergen elimination and AIT (96, 97, 107).

### 6.1 Allergen-specific immunotherapy

AIT is a specific desensitizing immunological method for treatment of allergic conditions, which consists of administering gradually increasing doses of a food allergen (food extracts or purified and sometimes modified food allergens) to the patient until desensitization is reached. This means switching the synthesis of allergen-specific IgE to immune IgG (3, 52, 96, 97). AIT is carried out only when the disease is based on the IgE-mediated mechanism, and a food product is vital (for example, milk allergy in children). AIT represents the only therapy capable of inducing a state of immune tolerance and provides potential to affect a sustained clinical benefit with long-lasting clinical remission of the allergic condition. In addition, AIT offers the possibility of preventing development of new food allergen sensitivities, inhibiting progression of food allergies toward allergic rhinitis and asthma, and improving a patient’s quality of life and lowering medication requirements. Currently, various routes of administration of food allergens are under evaluation, including oral, sublingual, and epicutaneous (6, 52, 62, 63, 91, 96–103). Research and clinical trials over the past few decades have elucidated the mechanisms underlying AIT-induced tolerance, involving reduction of allergen-specific TH2 cells, induction of regulatory T and B cells, and production of IgG and IgA blocking antibodies. To better harness these mechanisms, novel strategies are being explored to achieve safer, more effective, more convenient regimens and more durable long-term tolerance. These include alternative routes for current AIT approaches, novel adjuvants, use of recombinant allergens (including hypoallergenic variants), and combination of allergens with immune modifiers or monoclonal antibodies targeting the TH2 cell pathway (96–103). Although there have been many experimental and clinical trials, there is no consensus on criteria for assessing clinical and immunological outcomes of research, which is a severe obstacle to widespread adoption of these new treatments, and their effectiveness requires further study.

### 6.2 Oral immunotherapy

OIT is an emerging treatment for food allergies. Food allergy patients ingest increasing doses of allergens to desensitize their immune system and train it to not react to the problem food. OIT typically starts with very small doses of food allergens consumed under medical supervision. These doses are increased every one to several weeks until a small tolerated dose is reached that can be taken each day for months or years. Typical OIT doses of food proteins are measured in milligrams or grams. The food allergies treated with OIT in clinical trials included allergies to milk, egg, peanut, tree nut, wheat, soy, and sesame, as well as baked milk and baked eggs (97, 98, 103). Results of these researches show that OIT can be effective in inducing desensitization and, in some people with IgE-mediated food allergies, can induce sustained unresponsiveness (remission). In January 2020, an OIT treatment for peanut allergies received approval from the U.S. Food and Drug Administration (FDA) (97). However, this method is not routinely recommended for patients with food allergies due to uncertainty about outcomes including safety, long-term effectiveness, and overall impact on quality of life.

### 6.3 Sublingual immunotherapy

In order to undergo SLIT, patients must have a documented IgE-mediated food allergy, such as food allergies to peanuts, eggs, or milk. In the SLIT method, food allergens are dissolved in a small amount of liquid (usually this is a glycerinated allergen) and held under the tongue for several minutes before being spat out or swallowed. The procedure is repeated every day. SLIT doses are increased during an escalation phase until a consistent daily maintenance dose is reached. Compared to OIT, SLIT uses smaller doses because the amount of liquid that fits under the tongue is limited. SLIT doses are usually measured in micrograms or milligrams of protein. SLIT has been studied in treatment of hazelnut, peach, apple, milk, and peanut allergies with a substantial focus on treatment of peanut allergies. Phase II studies have shown that SLIT for treatment of peanut allergies increases the tolerated dose of peanut by a substantial margin with fewer and less severe side effects than other modalities. Long-term SLIT has been shown to induce sustained unresponsiveness, and there is evidence that high-dose SLIT protocols can achieve tolerance that approximates that of OIT. However, the cost of allergenic extract may make long-term, high-dose SLIT prohibitive. Therefore, some allergists have used food allergy SLIT as a temporary bridge to OIT (99, 100). Because long-term maintenance dosing regimens for food allergy SLIT have not been standardized, studies are needed to determine the minimum effective doses and duration of food allergy SLIT for various foods.

### 6.4 Epicutaneous immunotherapy

Epicutaneous (on the skin) immunotherapy, or EPIT, exposes tolerance-promoting immune cells in the skin to an adhesive dermal patch containing a small (micrograms) dose of food protein. Patches are being developed to treat peanut, milk, and egg allergies. EPIT starts with a small initial dose that is increased over time by wearing the patch for longer periods of day until a maintenance dose is reached in which each patch is worn 24 hours and replaced daily. EPIT has been tested in clinical trials for children with peanut allergies for its safety and efficacy in inducing desensitization (101–103).

### 6.5 Early introduction of allergenic foods in infancy

Studies support the existence of a critical time early in infancy during which the genetically predisposed atopic infant is at higher risk for developing allergic sensitization to food allergens. Therefore, dietary interventions in the first years of life have been analyzed for their effects on prevalence of allergic diseases including food allergies. Several observational and interventional studies demonstrated potential effectiveness of the early allergic food introduction strategy in prevention of food allergies, although strong evidence from randomized controlled trials is lacking and, sometimes, contrasting. Nevertheless, for children considered at high risk of developing food allergies the evidence for early introduction of allergenic foods, and in particular peanut and egg, is robust. The consensus exists that not only should such foods not be delayed, but that they should be introduced at approximately 4 to 6 months of age in order to minimize the risk of food allergies. Thus, currently, prevention strategies for food allergies have shifted from avoidance of foods to the early introduction of high-allergenic foods. American and European allergy expert committee guidelines as well as some other organizations recommend that solid foods should be introduced between four to six months of age in all infants (104–106). However, further studies are required to provide patients with evidence-based best practices.

### 6.6 Elimination diet

The elimination diet is considered the most effective modern concept for treatment and prevention of food allergies. An elimination diet is characterized by eliminating significant food allergens and replacing highly allergenic foods with low-allergenic or non-allergenic foods. An elimination diet requires exclusion from the diet not only of a specific food product responsible for the development of sensitization but also of any other foods in which it is included, even in trace amounts. For most patients, strict avoidance of confirmed food allergens remains the recommended standard of care. At this, of great importance is the appointment of adequate rational nutrition, corresponding in volume and ratio of food ingredients to the patient’s age and weight, concomitant somatic diseases, and other factors (6, 8–10, 19, 107).

### 6.7 Pharmacotherapy

Pharmacotherapy of food allergies aims to eliminate the symptoms of the existing disease and prevent exacerbations. Histamine is one of the most critical mediators responsible for developing clinical signs of food intolerance in true food allergies and pseudoallergies. Histamine has a wide range of pharmacological actions. It can influence pathophysiological reactions from various organs and systems: the respiratory tract (mucosal edema, bronchospasm, and mucus hypersecretion), the skin (itching, blistering-hyperemic reaction), the alimentary canal (intestinal colic, stimulation of gastric secretion), the cardiovascular system (expansion of capillary vessels, increased permeability, arterial hypotension, and cardiac arrhythmias), and smooth muscles (spasm). The critical role of histamine in pathogenesis of most allergic diseases determines the widespread use of histamine H1 receptor antagonists (6, 10, 108–110). These drugs have a pronounced antipruritic effect, ability to almost instantly alleviate the symptoms of allergic and pseudoallergic reactions, and have different clinical forms that provide flexible dosing for different age categories of patients, starting from infancy (8, 108–110).

### 6.8 Anti-IgE treatment

Understanding pathways that underlie development of various forms of allergic reactions has opened up new possibilities for their treatment with new immunobiological preparations aimed at binding antigen-specific IgE or its receptors (93–95). For example, omalizumab (OmAb), a recombinant anti-IgE antibody, has already been approved for treating severe allergic asthma and chronic urticaria not controlled by conventional therapies (36, 93). In recent years, several other drugs specific against IgE have also appeared but it remains unclear whether they will also be used in clinical practice.

A favorable factor in food allergies is that with age, the tolerance of the body to most food allergens increases. It is believed that if the food allergy has not stopped to 5-year age then age-related tolerance should not be expected to develop further (9, 10).

## 7 Clinical application of enterosorbents

An effective measure to detoxify the body is an efferent (sorption) therapy aimed at accelerated removal from the body of xenobiotics, harmful metabolites, circulating immune complexes, mediators of allergic reactions, inflammatory cytokines, food and industrial toxins, decay products of helminths, and other toxic substances. Experience with this type of treatment, mainly in post-Soviet countries, has shown the feasibility of its use in the acute period of allergic diseases and to prevent recurrence of the disease. Today, enterosorption is a component of complex therapy for various types of intoxication, including allergic, with a scientifically based and clinically proven efficacy (23–35, 111–125).

**Figure 3:**
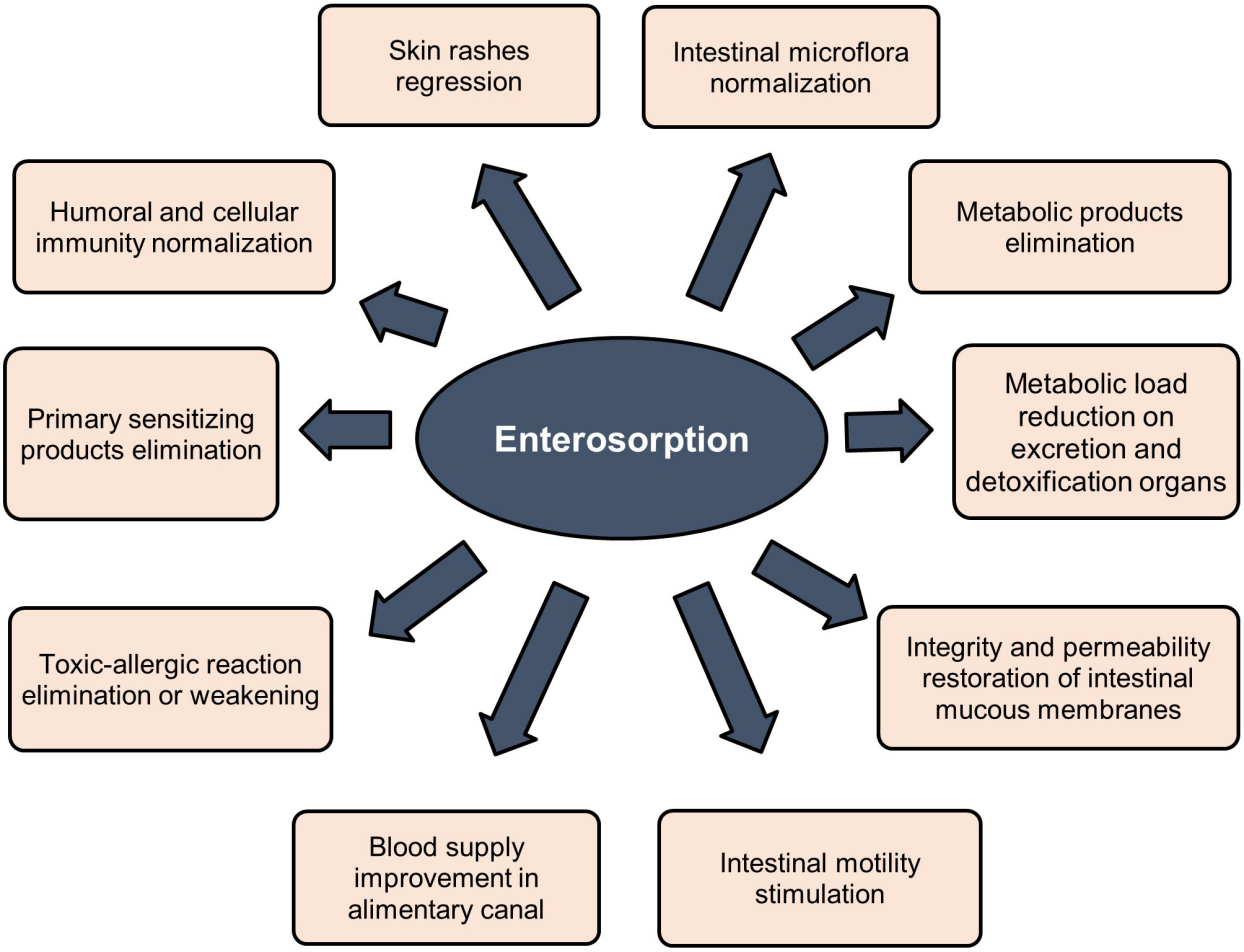
Clinical effects of enterosorption.

Sorbents are used to fix and remove food allergens from the digestive canal, in addition to products of incomplete enzymatic cleavage (medium molecular weight toxic metabolites) released as a result of allergic or eosinophilic inflammation, biologically active substances (serotonin, histamine, bradykinin, neuropeptides, prostaglandins, leukotrienes, etc.), pathogenic, opportunistic microorganisms and viruses, bacterial endotoxins associated with impaired intestinal microbiota. Binding of these substances in the intestinal lumen prevents their absorption, promotes their rapid and safe excretion, and improves conditions of the digestive system (23, 25–30, 34, 35). Indirect effects of sorbents are elimination or weakening of toxic-allergic reactions, prevention of endotoxicosis, reduction of the metabolic load on organs of excretion and detoxification, restoration of the integrity and permeability of the intestinal mucosa, stimulation of intestinal motility, and improvement of blood supply (24, 27–33, 120, 121, 124, 125). **Figure 3** summarizes clinically significant effects of enterosorbents application. The main advantage of enterosorption is its non-invasiveness, a small number of contraindications, the absence of complications, and changes in the biochemical composition of the blood through a sustained course of treatment (26, 30, 32, 116–118, 120, 121, 123). Enterosorbents are successfully used not only as a pathogenetic but also as an etiotropic mono- and combined therapy for intestinal infections and other infectious diseases (23, 26, 29, 30, 35). The clinical efficacy of some enterosorbents in mild and moderate forms of intestinal infections is not inferior to antibiotics (32). Effectiveness of enterosorption is comparable to the use of probiotics (120, 121).

There are several sorption methods using different physiological mechanisms. In practice of allergists, the most acceptable method is enterosorption, based on binding and removal of toxic substances and metabolites from the digestive canal (33, 111, 112). The gastroenterosorption detoxification involves the possibility of reverse passage of toxic substances from the blood into the intestine with their further binding by enterosorbents (9, 24, 30) The use of enterosorbents is applicable both in acute periods of allergic diseases and in inter-recurrent periods. Since the barrier function of the gastrointestinal canal mucosa is impaired, many toxic substances can be absorbed and lead to endotoxicosis, which is often found in patients with urticaria, atopic dermatitis, and other allergic reactions (24, 32, 33).

Enterosorption, as an effective detoxification method, is used for treatment of urticaria, psoriasis, atopic dermatitis, eczema, lupus erythematosus, dermorespiratory and dermointestinal syndromes, bronchial asthma, and other diseases (23–28, 32, 33, 114, 115, 119). With food and drug allergies and atopic dermatitis, using sorbents in complex therapy leads to more rapid regression of skin rashes and subjective sensations, as well as normalization of the gastrointestinal canal function. There is a positive effect in terms of laboratory parameters such as the decreasing number of eosinophils and level of total IgE in blood (28, 32, 119), increasing in cellular and humoral immunity, in particular, the number of T lymphocytes increases, eosinophilia decreases, level of circulating immune complexes decreases, while content of immunoglobulins class M and E stabilizes, itching reduces, and edema phenomena and hives decrease (24, 28, 33, 34, 113–119, 122). It is significant that at the same time, enterosorbents increase sensitivity to hormones, making it possible to reduce the amount of glucocorticosteroid therapy by 50 percent on average and, in some patients, complete withdrawal of hormonal therapy (32).

In patients with bronchial asthma, enterosorption has demonstrated positive results when combined with unloading and dietary therapy: signs of intoxication, intensity of concomitant skin-allergic manifestations, hormone dependence, drug intolerance, frequency and severity of bronchospasm attacks decrease (24, 33). The use of enterosorbents is effective in metabolic disorders in allergic patients and in detoxification of organs. Function of internal organs is improved with concomitant pathology of the hepatobiliary system, kidneys, cardiovascular vascular diseases, and diabetes. The ability of enterosorbents to reduce antigenic load on the body makes them suitable as a preventative measure for persons who come into contact with a large number of harmful substances in manufacturing and everyday life, as well as those living in industrial regions (23–35, 113, 115).

Development of intestinal dysbiosis plays an important role in formation of allergic reactions, in particular in food allergies. Dysbiosis contributes to increasing the permeability of the intestinal wall for products with sensitizing activity. Also, metabolic products of microorganisms themselves can be allergens. Therefore, normalizing intestinal microbiota, including the use of enterosorbents, is the fundamental principle of food allergies treatment (9, 29, 33). When the intestinal barrier function is intact, with typical microflora composition, endotoxins penetrate bloodstream in small amounts and are detoxified in liver hepatocytes. Conversely, with dysbiosis development, endogenous intoxication is maintained, resulting in increased permeability of the intestinal wall and weakening of adaptive and protective mechanisms. At the same time, some pathological processes in the body can cause intestinal dysbiosis and formation of endogenous intoxication by themselves, thus creating a vicious circle. These are diseases of the gastrointestinal canal with impaired motor evacuation and secretory functions (chronic pancreatitis, cholelithiasis, chronic gastritis, liver steatosis, hepatitis, etc.), resection of the stomach, small or large intestine, diverticular disease, past intestinal infections, helminthic infestations, giardiasis, or taking certain medications, including antibiotics, as well as chronic diseases of other organs and systems. In all these cases it is also advisable to prescribe enterosorption therapy (23–29, 35, 84, 100–105, 113–115).

Enterosorbents should be included in complex therapy in the first days or hours of exacerbation of an allergic disease or an acute allergic reaction. They are introduced into the gastrointestinal cavity orally. Usually, sorbents are prescribed 1.5-2 hours before meals. This period is needed for the enterosorbent to react with the stomach content and to be partially evacuated to the intestine, where its interaction with components of the intestinal contentcontinues. Sorbents and other drugs should not be applied simultaneously; there should be 2-3 hours between respective intakes. For most sorbents, the daily therapeutic dose is 0.2-1 g/kg of body weight, and maximal doses - are up to 2 g/kg of body weight. At the same time, the daily dose of enterosorbents is evenly distributed into 3-4 doses between breakfast, lunch, and dinner. The course of treatment is 6-8 days (no more than 14 days) with a gradual dose reduction over the last 2-3 days (23–30, 113, 115, 119, 120, 123).

Preventive (anti-relapse) dose of enterosorbents is 0.2-0.5 g/kg of body weight. Sorbents should be used for a 7-10-day period, during which it is possible to take them both in the morning and in the evening, 1.5-2 hours after dinner. A prophylactic course for allergic patients should be carried out monthly (in the first three months), then once a quarter during a year. Frequency of prophylactic enterosorption treatment is decided individually for each patient, taking into account the severity of both underlying and concomitant pathologies (24–27, 29).

Clinical effect of enterosorbents depends on timelines of their prescription - the earlier the drug is administered, the higher the sorption coefficient and clinical effect. A negative factor of several sorbents is, however, the sorption of vitamins, mineral salts, and other valuable substances, as well as the nonspecific sorption of enzymes (pepsin, trypsin, amylase), which in some cases requires correction by enzyme replacement therapy (26, 27, 32, 123).

General contraindications for the use of enterosorbents are erosive gastritis, peptic ulcer of the stomach and duodenum, ulcerative lesions of the intestine, and its atony. Cautious use of enterosorbents is recommended for patients with reduced intestinal peristaltic activity. A relative contraindication is a tendency to constipation. Excretion during the process of enterosorption should be daily; if necessary, laxatives are prescribed. While taking enterosorbents, increasing the drinking regimen and consuming foods with a high fiber content are recommended. Vitamin preparations are also recommended (24, 26–29, 32, 33, 35, 113–115, 119–121).

Sorption therapy of persons with allergic diseases should be individualized for each patient, considering comorbidity and tolerance to the sorption preparation. Mandatory indications for including enterosorbents in the complex of rehabilitation measures are acute allergic reactions of any etiology. Enterosorbents are indicated for prophylactic purposes for people with hereditary atopy and for patients receiving long-term corticosteroid therapy with concomitant liver, kidneys, and intestines pathology that slows down the detoxification of xenobiotics. Sorption methods for treating allergic diseases and concomitant pathological conditions are highly effective and practice safe methods of endogenous detoxification. They are recommended for widespread introduction into clinical practice in inpatient and outpatient settings for different age groups of patients (9, 24, 33, 113–119, 123). In post-Soviet countries, enterosorbents are included in the protocols for treatment of various manifestations of food allergy in children, along with an elimination diet and systemic treatment, depending on clinical symptoms of the disease (24–26, 29, 32, 33).

For enterosorbents, the main considerations that should be taken into account, especially for enterosorbents used in pediatric practice are the following:

- lack of toxicity;
- good evacuation from the stomach;
- no damaging effect on the gastrointestinal canal;
- high sorption capacity with minimal loss of valuable ingredients;
- as they pass through the intestine, the bound components should not be desorbed and should not change the medium pH;
- convenient form and ease of dosing;
- good organoleptic properties;
- the sorbent should favorably influence the secretion processes and the intestinal microbiota (23, 32, 111, 112, 126, 127).

Many enterosorbents that meet the above requirements already exist in the arsenal of therapies available to pediatricians. These methods have been used in clinical practice for over 30 years and are used globally (23–33, 111, 112, 128–132).

**Figure 4:**
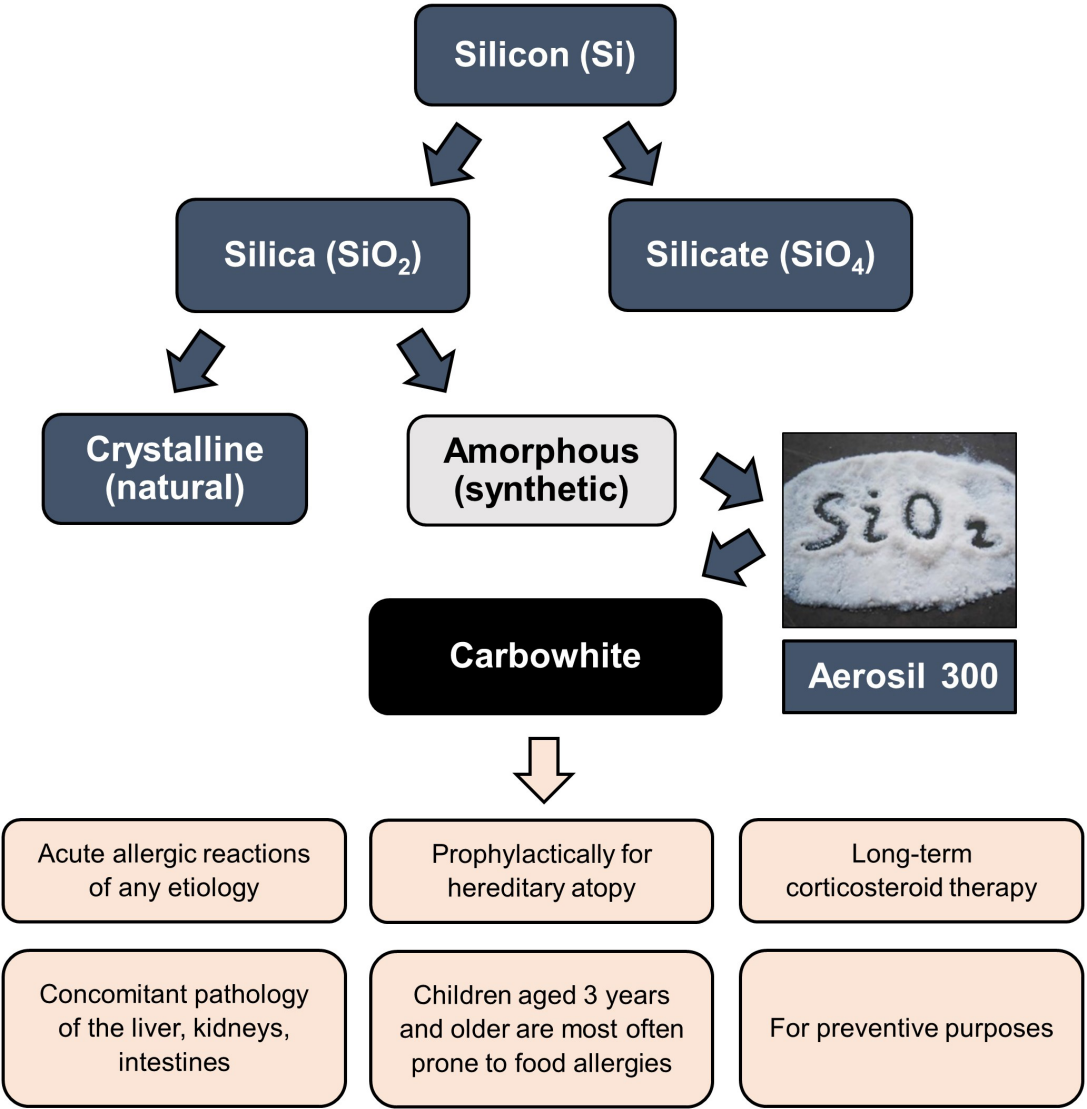
The place of White Coal (Carbowhite) in the structure of silicon derivatives and indications for its use. The active substance of White Coal is Aerosil 300, produced by the company Evonik (Germany) - patented the highly dispersed non-porous anhydrous amorphous colloidal silicon dioxide raw material (silica, SiO2), having a powdery or granular structure of white color.

All of them have certain advantages and can be prescribed to allergic patients depending on individual indications, including such characteristics as tolerability, efficacy, features of the course of the underlying disease, and presence of concomitant diseases. This review is limited to detailed consideration of enterosorbents based on silicon dioxide, particularly a drug known under the trade name “White Coal” (Carbowhite) produced by the Ukrainian company OminiFarma (**Figure 4**).

## 8 Silicon dioxide and silicon dioxide-based enterosorbents

Silicon (Si) is one of the chemical elements found in the Earth’s crust, and it ranks second in abundance after oxygen (125, 133). The oxide forms of silicon are silicate (SiO4) and silica (silicon dioxide, SiO2), which are also present in various plants, organs, and tissues of humans and animals, and enter the human body with food, being physiological components of the body (125, 133). Silicon is widely used in industry, and its oxide forms are often used in biomedical applications. Silica nanoparticles have several rare properties, such as ease of synthesis, modifiable surface, strong mechanical properties, and relatively inert chemical composition, which ensure the durability of biomaterials based on them (134–137).

There are two primary states of silica - crystalline and amorphous (**Figure 4**). Both conditions have the same molecular formula but differ in structural arrangement (133, 134). The lattices of crystalline silica are arranged regularly, while the lattices of amorphous silica are arranged irregularly (121–139).

Crystalline silica has several forms. A well-known form is α-quartz, which can be converted to β-quartz, tridymite, and cristobalite when heated. There is also porous crystalline silica called porosil; all porosils are synthetic. Terms nanoporous, mesoporous, and microporous refer to the pore diameter. “Nanoporous” refers to materials with pore diameters less than 2 nm, “microporous” refers to materials with pore diameters greater than 100 nm, and “mesoporous” refers to materials with pore diameters between 2 and 100 nm (133, 134, 136, 140). Mesoporous silicon and silica particles are ideal candidates for controlled drug release due to their rare properties, such as high surface area, large pore volumes, controlled pore sizes, and good chemical and thermal stability (135, 136, 141).

Amorphous silica can be divided into three groups: natural form (rare), a by-product of power plants and metallurgical production, and synthetically created silica (133, 134). Amorphous synthetic silica is a promising candidate for gene carrier and molecular imaging, mainly due to its highly tunable biocompatibility and stability (126, 127). In addition, it is also used in dietary supplements (142), catheters, implants, and dental fillers (143).

Nanoparticles are one of the most general terms for designating isolated ultradispersed objects, duplicating previously known terms (colloidal particles, ultradispersed particles) but differing from them in clearly defined dimensional boundaries. Solid particles less than 1 nm in size are usually referred to as clusters and more than 100 nm - to submicron particles. A nanoparticle is an isolated solid-phase object, the dimensions of which in all three sizes range from 1 to 100 nm. At the same time, in some fields of knowledge, in particular, in biomedical nanotechnologies, nanoparticles are often conventionally referred to as objects with a diameter of up to several hundred nanometers, the small size of which also plays a significant role in their properties and application, in particular, providing increased mucosal absorption during oral administration. At present, it is generally accepted by the world scientific community that nanoparticles of synthetic amorphous silica and, especially, submicron particles do not have toxic side effects when used in sufficiently high doses and are almost eliminated from the gastrointestinal canal physiologically without entering into biological fluids (23–30, 32, 33, 91, 92, 114, 133–136, 138, 144–151). Moreover, the use of amorphous silica nanoparticles (Aerosil 380 and Aerosil 200) has been approved in the European Union as a safe food additive E551, and Aerosil 300 is widely used in pharmaceutical and cosmetic industries (135, 144, 145, 151–153).

The Manufacturers Association of amorphous silica and independent studies have verified the safety of synthetic amorphous silica. In particular, in several works, based on a comparison of numerous clinical and laboratory studies, it was shown that none of the forms of silica, including colloidal nanoparticles, bioaccumulates. All of them are eliminated from living organisms within a short time using physiological mechanisms (150, 151). Research centers and individual experts in the European Union and the USA have evaluated the daily dosage limits of synthetic amorphous silica, which would lead to undesirable toxicological effects. A brief review of animal studies on the oral safety of amorphous silica has been published by the Organization for Economic Co-operation and Development (OECD) (152) and the European Center for Ecotoxicology and Toxicology of Chemicals (ECETOC) (153).

Silicon dioxide-based enterosorbents are produced from high-quality patented raw materials from Evonik (Germany), obtained using a unique technology that allows the creation of soluble porous and non-porous materials from the lightest fraction of silicon dioxide (nanoparticles 7-40 nm in size), which include Aerosil Ox50, Aerosil 130, Aerosil 150, Aerosil 200, Aerosil 300, Aerosil 380 (135–137, 144, 152, 153). They have a high degree of dispersion and a large sorption surface area. Therefore, the daily dose of silica preparations is only 2-4 g, almost ten times less than that of activated carbon (29). Silica preparations are prescribed for adults and children over three years old, 3-4 tablets up to 4 times daily. With prolonged administration in therapeutic doses, silicon dioxide preparations do not cause noticeable changes in the activity of enzymes of the intestinal mucosa; they differ in lower (compared to other sorbents) excretion of vitamins and microelements and their rapid recovery to normal levels without additional drug load (30, 32, 34, 35, 116, 118), which is typical, in particular, for the enterosorbent White Coal (Carbowhite).

## 9 White Coal (Carbowhite)

The active substance of the enterosorbent White Coal is a highly dispersed, non-porous colloidal anhydrous silicon dioxide (silica, SiO2). The drug is manufactured using modern, highly dispersed technology from patented raw materials, silicon dioxide Aerosil 300 from Evonik (**Figure 4**). The safe dosage range of Aerosil 300, used in the drug White Coal varies from 100 to 300 mg/kg of body weight/day, with short-term use - up to 1000 mg/kg per day (23).

The production technology guarantees that the White Coal tablet will disintegrate in the body in a few minutes, turning into a highly dispersed suspension, while 1 g covers 380-400 m^2^. The product was introduced to the Ukrainian market in 2008. To date, the clinical evaluation of the efficacy and safety of the product has been shown by multiple studies, which also showed the absence of any undesirable side effects of treatment (23, 35, 115–118, 154–160). White Coal is registered as a drug in Ukraine, Georgia, and Uzbekistan.

**Figure 5:**
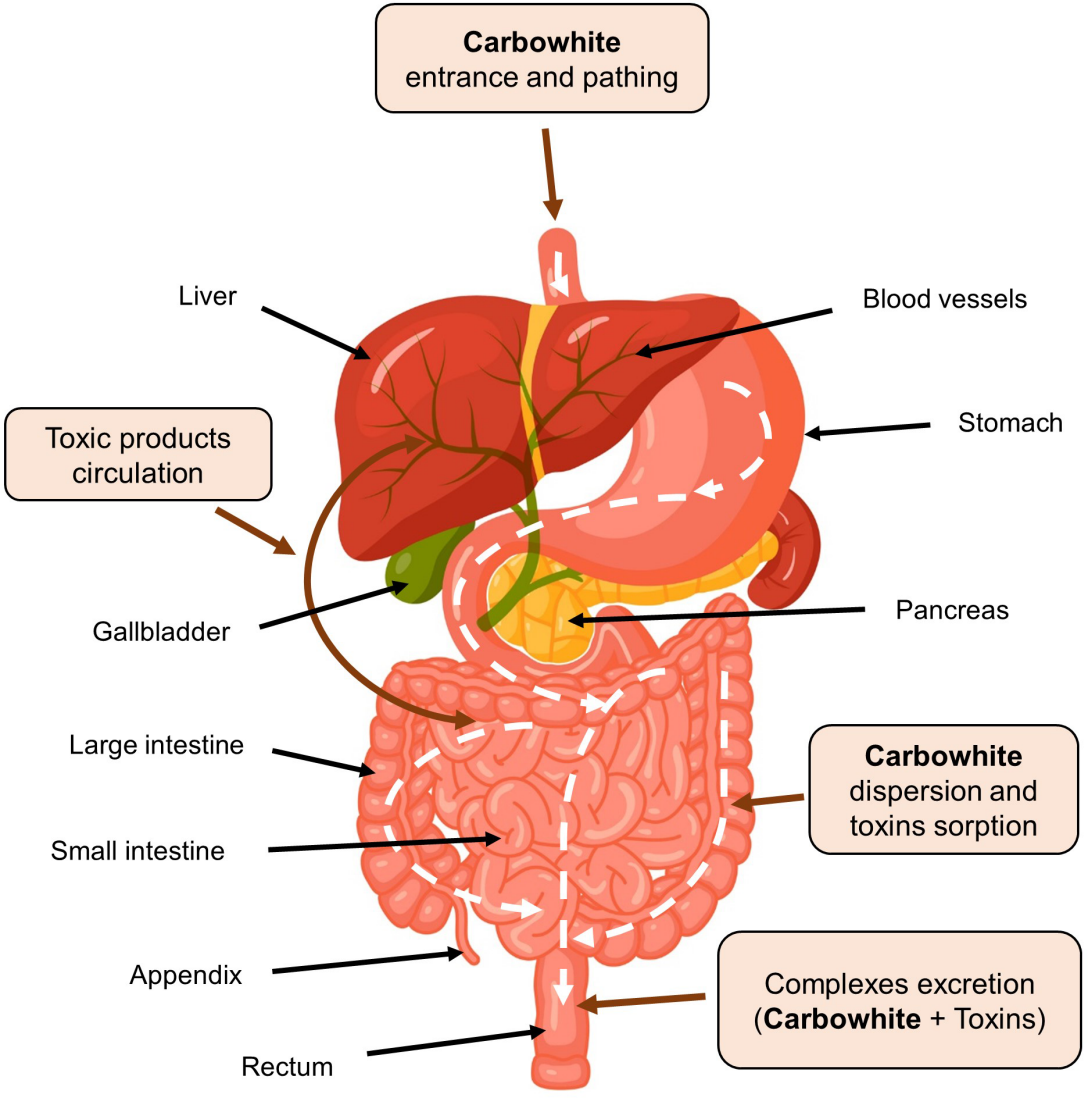
The mechanism of sorption and excretion of toxic products from the digestive system by enterosorbent White Coal (Carbowhite).

According to the manufacturing specification, the particles of silicon dioxide in feedstock used for production of the White Coal have a size of 7 nm, which during the production process under the influence of high temperature, form submicron agglomerates with a size of more than 100 nm (137, 160). It is essential that during the production of the White Coal, silicon dioxide Aerosil 300 is subjected to the process of further additional coarsening of agglomerated particles according to the patented wet granulation technology of the Omnifarma company, that is, the White Coal is not a nanoproduct and does not carry the risks associated with possible systemic toxicity or toxicity to the immune system when taking therapeutic doses of silicon dioxide nanoproducts (146–153, 161). No evidence exists of natural mechanisms for the degradation of Aerosil 300 agglomerates to their original nanoparticles in the human body. Such deagglomeration can only be achieved by ultrasonic irradiation (138) or by passage through the gastrointestinal canal with pH > 12 (151), which is not possible in the human body (162). Therefore, amorphous silicon dioxide used in White Coal is a structurally stable submicron agglomerate of nanoparticles that remain intact in the gastrointestinal canal. Moreover, these agglomerates cannot pass through the intestinal epithelial barrier and thus do not circulate in the body fluids with the exception possible presence of a very limited number of free nanoparticles. This unique property of White Coal significantly contributes to its safety profile when used as the enterosorbent and reduces potential negative consequences associated with the possible presence of trace amounts of free amorphous silica nanoparticles in the product. Nevertheless, to clarify the safety profile of the White Coal drug, it is advisable to conduct further studies of its pharmacokinetic and pharmacodynamic properties. White Coal is produced as tablets containing 210 mg of the active substance. It is easily dispersed in the gastrointestinal canal, forming a large sorption surface. It is almost entirely (more than 99%) excreted from the intestine through feces, practically without entering the systemic circulation (less than 1%). From systemic circulation, the drug is excreted through the kidneys with urine. The enterosorbent is widely used as mono and complex therapy for acute intestinal diseases (salmonellosis, food poisoning), acute diarrhea of various etiologies, and exogenous intoxication with household and industrial toxins, drugs, alcohol, and food intoxications. It is also used as an adjuvant for viral hepatitis, allergic diseases, dermatitis, late gestosis of pregnant women, cirrhosis of the liver, chronic toxic hepatitis, chronic non-calculous cholecystitis, non-alcoholic fatty liver disease and other mono and complex pathologies (**Figure 4**). At the same time, the enterosorbent does not cause constipation and absorbs toxic substances in the intestines selectively, without loss of beneficial trace elements. In addition, due to the formation of concentration and osmotic gradients, White Coal promotes the removal of various toxic products from the internal environment of the body (blood, lymph, interstitium), including medium molecules, oligopeptides, amines, and other substances, which are formed in a result of systemic inflammatory and allergic reactions (23, 35, 113, 115–118, 154–161). **Figure 5** demonstrates the distribution and the mechanism of action and evacuation of the enterosorbent White Coal from the digestive system.

A remarkable feature of enterosorbent White Coal is the combined effect of silicon dioxide (Aerosil 300) and microcrystalline cellulose, which is part of its composition as an additional active substance. Microcrystalline cellulose positively impacts the tablet’s properties (163) and functions of the gastrointestinal canal (164). This food additive enhances the buffering effect of food, potentiates the hydrolysis of proteins in the stomach, modifies the secretion of gastrointestinal hormones, affects the transit and hydrolysis of nutrients in the small intestine, inhibits the absorption of monomers and bile acids, reduces intracavitary pressure in the colon, stimulates intestinal motility and growth of microflora, reduces the risk of gallstone formation, and has a hypoglycemic effect (29, 31, 164). Following a request from the European Commission, the EFSA Panel on Food Additives and Nutrient Sources added to Food (ANS) delivered a scientific opinion re‐evaluating the safety of microcrystalline cellulose (E 460(i)) and modified variations. From the reported studies, the Expert Panel considered a safe application for the microcrystalline, powdered, or modified cellulose to be around 660–900 mg/kg body per day (165).

In 2016, a multicentre, randomized, double-blind, placebo-controlled study of the efficacy and safety of White Coal in acute diarrhea was conducted (level of evidence I (A) according to CEBM (35). Administration of White Coal to patients (children and adults) with acute diarrhea during 3-5 days in doses 1260, 1890, and 4200 mg daily (for different groups) resulted in the termination of diarrhea in an average of 1.7 days without any side effects (35). The clinical trial results were approved by the US FDA - available at the ClinicalTrials.gov web resource (166).

In 2009, the results of a clinical study on the use of the enterosorbent White Coal in the complex treatment of children with manifestations of food allergies were published (113). The study was conducted as an open randomized in the National Children’s Specialized Hospital “Okhmatdet” (Kyiv, Ukraine). Under observation were 46 children aged 1 to 15 years with manifestations of food allergies against the background of various allergic pathologies.

The studies were carried out in dynamics: in the acute period of the disease (before treatment), on the 7th and 14th days of complex therapy with the inclusion of the White Coal. The enterosorbent was administered orally in 3 doses at a total daily dose of 150 mg/kg, 1 - 1.5 hours before meals and between doses of other drugs for 14 days. The effectiveness of treatment was assessed according to the following criteria: SCORAD index, level of total and specific IgE, degree of dysbiosis, level of hematological activity, the full dose of systemic glucocorticosteroids, and duration of the acute period.

**Figure 6:**
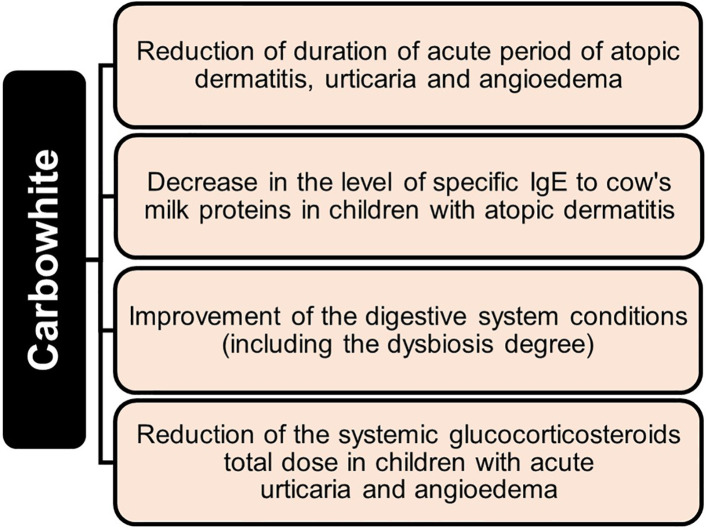
Clinical effectiveness of a 14-day course of using enterosorbent White Coal (Carbowhite) in children aged 1 to 15 years with manifestations of food allergies of various etiologies.

The treatment resulted in a reduction in duration of the acute period of atopic dermatitis, urticaria, and angioedema, a decrease in the level of specific IgE to cow’s milk proteins in children with atopic dermatitis, and an improvement in the state of the gastrointestinal canal with the decreasing of dysbiosis symptoms. The total dose of systemic glucocorticosteroids was reduced in children with an acute episode of urticaria and angioedema. These data indicate a relatively high clinical efficacy of the 14-day course of the enterosorbent White Coal in children aged 1 to 15 years with manifestations of food allergies against the background of various pathologies (**Figure 6**).

## 10 Nanoparticles-associated silica potential risks

The effects of silica exposure on the body, especially crystalline silica, have been studied extensively. Studies have shown that exposure to crystalline silica associated with the use or production of this material causes silicosis (fibrous lung disease) in workers and that this exposure is also associated with other lung diseases such as lung cancer, lung emphysema, pulmonary tuberculosis, autoimmune diseases (149, 166–169). The toxicity and pathogenicity profile of silica, and therefore the risk of silicosis, depends on the inhaled particles’ size and physical and chemical properties (167–171). It has long been thought that the crystal structure imparts silica toxicity. Still, recent studies show that the number and distribution of sialon and siloxane groups, rather than crystallinity, act as primary toxic factors (172, 173).

The safety of using amorphous silica and, mainly, silica nanoparticles, their systemic toxicity, and toxicity to the immune system are also discussed (133, 146–153, 161). Amorphous silica used to be considered less harmful than crystalline silica. However, recent studies have shown that amorphous silica nanoparticles may have the same potential toxicity as crystalline ones (146, 173). It is believed that the physicochemical properties of amorphous silica nanoparticles can have various toxic effects *in vitro* and *in vivo*. Several reviews have considered this issue in detail (134, 161, 173, 174).

The studies were mainly focused on the initial stages of the interaction of silica with cells located in the lungs and immunological modifications of the innate immune cellular response. Special attention was paid to the primary interaction of silica with alveolar macrophages. Usually, silica particles enter the alveolar space after inhalation and interact with macrophages, resulting in inhaled particles entering the phagosomes. The macrophage receptor MARCO has been described as the primary molecule responsible for silica recognition and uptake by macrophages (175). Other receptors from the group of recognition receptors (Pattern receptors) may also be involved in this process. In particular, it has been shown that CD204 can interact with silica and inhibit the activity of macrophages. In addition, significant downregulation of Toll-like receptor 2 (TLR2) after silica exposure may contribute to increased susceptibility to tuberculosis (176). Finally, the inability of macrophages to digest and remove phagocytosed silica particles leads to persistent inflammation and modification of the cellular response (177).

Some authors believe silica nanoparticles can interact with immunocompetent cells and cause immunotoxicity (146, 176, 177). The effect of silica nanoparticles on the immune system depends on the physicochemical properties of the nanoparticles and type of cells they affect and lead to different results (134, 138, 178, 179). The effector cells involved in these reactions are mainly tissue macrophages, peripheral blood monocytes, polymorphonuclear leukocytes, dendritic cells, lymphocytes, mast cells, and other cell types, modifying the immune response (34, 134, 146–149, 161, 178, 179).

Silica exposure can reduce cellular function and DCs’ activation, leading to a nonspecific attenuated inflammatory response disrupting antibacterial defense mechanisms and increasing susceptibility to bacterial infections, primarily Mycobacterium tuberculosis and other mycobacteria (176, 180, 181). However, the impact of silica on immune responses is complex and does not always result in increased susceptibility to bacterial infections; some effects promote the elimination of mycobacteria rather than their reproduction (161).

Potential toxicity of nanoparticles to immune cells is related to their ability to induce direct cell damage, such as apoptosis and necrosis. Functions of immune cells may change after interaction with nanoparticles, and this may affect immunospecific signaling pathways. Toxic effects can be assessed by functional activity of cells, development of pro-inflammatory reactions, generation of reactive oxygen species, cellular dysfunction, cytotoxicity, genotoxicity, and their underlying mechanisms (134, 182–191). Assessing the interaction between nanoparticles and cells of the immune system is critical in determining safety of silica nanoparticles, which are widely used in biomedical applications due to their unique chemical and physical properties (192, 193). In general, toxic effects of silica nanoparticles on the immune system *in vivo* are rare and occur only at oral doses significantly higher than the recommended allowable values (134, 170–174, 182–184, 194). Based on risk assessments of amorphous silica toxicity with known sources of silica, such as dietary supplement E551, a safe upper intake level has been estimated at 720 mg/day for a 60 kg adult, equivalent to 12 mg silica/kg body weight/day (133).

Considering the discussion regarding the possible toxicity and immunotoxicity of amorphous silica nanoparticles, the expert committee of the European Commission in 2018 revised the recommendations regarding the food additive E551. It concluded that, at the moment, there are no grounds for conclusions about the toxicity of this product for humans, and the recommendations of the European Commission made for it earlier, in 2009, remain valid (144, 145).

## 11 Conclusion

Amorphous silicon dioxide food supplements and drugs, particularly White Coal (Carbowhite), are safe at recommended doses because they are structurally stable submicron agglomerates of nanoparticles that do not disaggregate in the gastrointestinal canal and are hardly absorbed into body fluids. Given the composition and properties of White Coal, its usage scope can be extended to a wide range of allergic diseases, primarily food allergies. It is advisable to include White Coal in complex treatment of food allergies in adults and children over three years of age, as it contributes to faster relief of symptoms of damage to the gastrointestinal system, reduces endogenous intoxication, reduces drug load, and improves the patient’s quality of life. In addition, the high safety profile of White Coal and its unique sorption properties make it possible to recommend this enterosorbent to pregnant and breastfeeding women, especially during an exacerbation of allergic inflammation, to prevent allergic sensitization in hereditarily predisposed newborns.

## Author contributions

VS collected and reviewed the literature, wrote and redacted the manuscript, and designed the illustrations. OK and EO redacted the manuscript and illustrations. VC and DD revised the manuscript and figures, contributed to rewriting Section 6 “Treatment of Food Allergy”, approved the final version of the revised manuscript, and agree to be accountable for all aspects of the work. All authors contributed to the article and approved the submitted version.
